# Advances in Neuromodulation and Digital Brain–Spinal Cord Interfaces for Spinal Cord Injury

**DOI:** 10.3390/ijms26136021

**Published:** 2025-06-23

**Authors:** Phillip Jaszczuk, Denis Bratelj, Crescenzo Capone, Marcel Rudnick, Tobias Pötzel, Rajeev K. Verma, Michael Fiechter

**Affiliations:** 1 Interdisciplinary Spine Center, Luzern Cantonal Hospital, University of Luzern, 6000 Luzern, Switzerland; 2 Spine Surgery, Swiss Paraplegic Center, 6207 Nottwil, Switzerland; denis.bratelj@paraplegie.ch (D.B.); crescenzo.capone@paraplegie.ch (C.C.); marcel.rudnick@paraplegie.ch (M.R.); tobias.poetzel@paraplegie.ch (T.P.); michael.fiechter@paraplegie.ch (M.F.); 3 Swiss Paraplegic Research, 6207 Nottwil, Switzerland; rajeev.verma@paraplegie.ch; 4 Department of Radiology, Swiss Paraplegic Center, 6207 Nottwil, Switzerland

**Keywords:** spinal cord injury, neuromodulation, brain–computer interface, brain–spine interface, epidural spinal cord stimulation, neurorehabilitation

## Abstract

Spinal cord injury (SCI) results in a significant loss of motor, sensory, and autonomic function, imposing substantial biosocial and economic burdens. Traditional approaches, such as stem cell therapy and immune modulation, have faced translational challenges, whereas neuromodulation and digital brain–spinal cord interfaces combining brain–computer interface (BCI) technology and epidural spinal cord stimulation (ESCS) to create brain–spine interfaces (BSIs) offer promising alternatives by leveraging residual neural pathways to restore physiological function. This review examines recent advancements in neuromodulation, focusing on the future translation of clinical trial data to clinical practice. We address key considerations, including scalability, patient selection, surgical techniques, postoperative rehabilitation, and ethical implications. By integrating interdisciplinary collaboration, standardized protocols, and patient-centered design, neuromodulation has the potential to revolutionize SCI rehabilitation, reducing long-term disability and enhancing quality of life globally.

## 1. Introduction

Spinal cord injury (SCI) imposes profound physical, psychological, and socioeconomic challenges, with limited effective treatments to restore function. This review evaluates progress in neuromodulation and digital brain–spinal cord interfaces, which enable physiological walking by bridging neural lesions in SCI. We focus on the critical steps needed to transition these technologies from experimental clinical trials to practical, everyday clinical and surgical use, addressing scalability, patient selection, ethical issues, postoperative care, and current advancements. Unlike stem cell research, immune therapy, and other resource-intensive or ethically controversial approaches, neuromodulation has demonstrated rapid progress and tangible clinical outcomes, with potential for swift development and application [1,2]. The scalability and bench-to-bedside implementation depend on logistics, regulatory approvals, surgical refinements, acceptance, ethical standards, costs, and new industry and medical standards to deliver these technologies to patients safely and efficiently [3,4,5]. Successful adoption could significantly reduce neurorehabilitation costs, particularly for complete paraplegia, which incurs substantial expenses [6,7]. Standardized protocols are essential to alleviate the socioeconomic burdens of SCI, ensuring equitable access to therapy and improving quality of life at scale [7]. Practical, patient-oriented interdisciplinary care is vital to transform this technology into routine practice, potentially revolutionizing SCI rehabilitation [3,8]. The high biosocial and financial burden of SCI on patients, communities, and healthcare systems is particularly pronounced in low-resource settings, where enhancing neurological function could yield significant socioeconomic benefits [7]. Collaborations among academia, industry, and healthcare systems can position neuromodulation as a cornerstone of SCI treatment, reducing long-term disability and enhancing patient autonomy [9].

### 1.1. Fundamentals of Key Technologies

BCI technology enables individuals to control devices directly with their brain signals, bypassing the need for complete neuromuscular pathways, which is especially beneficial for those with SCI. BCIs work by recording neural activity through electrodes—such as those implanted in the brain or on the scalp—and translating these signals into commands for computers, prosthetics, or other systems, offering a way to restore communication or mobility by creating a direct link between the mind and external technology [2]. Epidural Spinal Cord Stimulation (ESCS) involves the use of implanted electrodes with impulse generator technology to deliver controlled electrical impulses to the spinal cord, modulating nerve activity to improve function, as demonstrated by Harkema et al., who showed that ESCS combined with locomotor therapy can enable voluntary movements and standing in patients with motor-complete paraplegia by enhancing the spinal cord’s natural plasticity and responsiveness [8]. The brain–spine interface (BSI) builds on these principles by integrating brain signal recording with spinal cord stimulation, creating a seamless connection to restore lost functions. This approach can enable voluntary movements in humans with chronic complete paralysis by coordinating neural signals between the brain and spine [2]. Together, these innovative technologies open new possibilities in neurorehabilitation, making advanced medical solutions more accessible to a wider audience by harnessing and enhancing the body’s neural networks.

### 1.2. Overview of the Important Challenges and Potential Gains of Bringing Brain–Spine Interfaces from Bench to Bedside

Important Topics:

- Integration of BSIs in SCI rehabilitation.
- Comparative analysis of leading implantable brain–computer interfaces (iBCIs);
- Advances in ESCS and adaptation of spinal neuromodulation technologies.

Key Challenges:

- Surgical complexity and need for specialized neurosurgical training.
- Regulatory hurdles for clinical translation of technologies and device approval.
- Signal degradation and long-term reliability of neural implants.

Clinical Implications:

- Potential to restore walking in patients with complete SCI.
- Scalable solutions for low-resource settings using minimally invasive systems.
- Frameworks for personalized neurorehabilitation combining artificial intelligence (AI), BSIs, and physical therapy.

## 2. Scientific Considerations

Progress in axonal regeneration for SCI treatment remains hindered by complex developmental challenges, including risky intrathecal applications, difficulties in obtaining clinical trial data, and limited understanding of pharmacokinetics [2,10]. In contrast, neuromodulation has achieved exponential advancements without requiring axonal restoration [11,12,13]. Medical research aligns with technological progress, potentially relegating regenerative strategies to augmenting neural interfaces and conduction capacity [14]. For instance, electrochemical neuromodulation therapy enhances volitional motor skills by reorganizing residual projections, strengthening supraspinal control of spinal circuits, possibly via cortico-reticulospinal pathways [4,5]. Targeting spared lumbar circuits and preserved neurons below the injury level addresses conduction failures and disrupted descending pathways to improve SCI recovery [4]. Insights from axonal regeneration research remain valuable for understanding neuromodulation’s potential in severe spinal cord lesions [15]. Complete injuries with near-total transections and limited residual pathways pose significant challenges across all SCI treatment approaches [16]. Studies in animal models using calculated injuries demonstrate neuromodulation’s ability to restore adaptive and interlimb control of paralyzed limbs in severe injuries [5,6]. The complex orchestration of locomotor muscle activation has been successfully replicated by implanting intracortical microelectrodes in lesioned primate models, paired with spatially selective epidural pulse generators and real-time wireless control, restoring weight-bearing locomotion [17,18]. These findings underscore neuromodulation’s capacity to bypass traditional barriers to central nervous system regeneration. Translating these successes to humans requires personalized approaches to address diverse injury profiles [6,17]. Integrating sensory information into stimulation protocols is crucial for improving outcomes and unlocking therapeutic potential [13,19]. The efficacy of brain–computer interfaces (BCIs) hinges on their ability to decode complex motor and fine motor intentions while integrating sensorimotor signals [20]. Clinical trials will require interdisciplinary frameworks to incorporate combinatorial therapies tailored to varied tetraplegic and paraplegic injury profiles, necessitating interprofessional translational research to integrate biomaterials and growth factors into neural interface augmentation [21].

## 3. Neurosurgical Considerations

Widespread adoption of neuromodulation and digital brain–spinal cord interfaces demands precise, safe electrode placement, durable implants, and real-time neural decoding systems. Intraoperative neuromonitoring ensures accurate electrode placement tailored to individual patient anatomy and injury characteristics, optimizing neuromodulation efficacy and minimizing off-target effects [22]. Long-term success relies on biocompatible materials to reduce tissue reactions and ensure device longevity [23]. Scalability necessitates standardized implant designs balancing cost and performance. Minimally invasive and advanced surgical techniques, such as neuro-navigation, can reduce surgical risks and recovery times, enhancing accessibility [24]. Neurosurgeons require specialized training, and regulatory frameworks must evolve to approve devices for broad use without compromising safety [8]. Synergistic use of imaging, myelin-associated inhibitor augmentation, real-time BCIs with electrical stimulation, and machine learning-driven neural decoding enhances surgical precision and long-term outcomes [22,25]. Human electrode array control has shown quantitative comparability to animal models, underscoring the need for global neurosurgical training programs, potentially using virtual reality tools to standardize expertise across diverse healthcare settings [8]. Epidural spinal cord stimulation (ESCS) electrode implantation, established for pain management and autonomic dysfunction, now supports neurological restoration in SCI proof-of-concept studies. This shift from compensation to functional restoration raises ethical considerations. Clinical trials must be robustly designed, with informed consent and ethical guidelines guiding surgeons, as patients weigh surgical risks against the intensive rehabilitation required for device-assisted outcomes [26].

Neurosurgical implantation of implantable brain–computer interfaces (iBCIs) faces challenges in implant reliability, placement precision, intra-axial stability, timing of intervention, and regulatory compliance across manufacturing and medical domains [27,28]. Ongoing clinical trials address these issues using robot-assisted electrode alignment to target cortical motor centers responsible for movement. Experience with Utah electrode arrays or electrocorticography (ECoG) grids highlights the need for meticulous craniotomy and durotomy to access cortical surfaces while avoiding vascular injury, with intraoperative neuromonitoring ensuring signal strength [22,27]. Novel intravascular approaches, such as endovascular stent–electrode arrays deploy electrodes via the cerebral vasculature, reducing surgical complications but requiring neuro-navigation to target sensorimotor cortex and introducing risks inherent to interventional neuroradiological procedures. Managing gliosis and scar tissue formation, which degrade signal quality over time, remains a significant challenge [23,27]. Biotechnological advancements, such as soft electrode designs and brain-compatible coatings, could mitigate reactive gliosis and scar formation, though their integration into operating theaters requires rigorous standardization [23,28]. Widespread adoption of these implants necessitates neurosurgical training, potentially via virtual reality platforms, to prepare surgeons for specific procedures [8]. Ethical neurosurgical practice demands a transparent communication of risks, including infection, device failure, or unanticipated neurological effects, aligning with informed consent principles, particularly in complex clinical trials [26,28].

## 4. Advancements in Implanted Brain–Computer Interfaces

Implantable brain–computer interfaces (iBCIs) represent the most exciting area of functional neurosurgery since the adoption of deep brain stimulation (DBS) into routine clinical practice. Neural decoding, in addition to informing us about the intricacies of various neural functions, can help us to evolve the field of neuromodulation to treat what were once thought of as permanent injuries to restore neurological function in SCI. This will be accelerated by electrodes with increasing sensitivity/density and improvements in design compatible with the concepts of keyhole or minimally invasive surgery, as well as by a maximal scalability of implementation benefits from surgery with lasting treatment effects. These advancements are pivotal for restoring motor function in SCI by decoding cortical signals and interfacing with spinal stimulation systems. Below, we analyze key iBCI devices, focusing on their technological developments, clinical outcomes, complication profiles, and relevance to SCI rehabilitation, with an emphasis on their integration into brain–spine interfaces (BSIs). The findings are summarized in Table 1.

### 4.1. Neurorestore WIMAGINE-Based Brain–Spine Interface

The WIMAGINE implant, developed by the Courtine group in collaboration with CEA/LETI and Clinatec, represents a foundational iBCI in the field of restoring locomotion in SCI. The key to its success is the decoding of motor intention to control spinal stimulation in real time. This wireless electrocorticography (ECoG) device has a 64-channel array encased in a biocompatible titanium housing and a 6-year preclinical safety record in non-human primates [29]. A critical landmark trial demonstrated the restoration of overground walking in a single subject (NCT02550522), with the help of bilateral WIMAGINE devices decoding cortical signals and triggering epidural spinal stimulation [27]. A rehabilitation period of two months was required with sustained improvement over two years, a remarkable accomplishment [27,30]. Decoding accuracy was documented at 80–90% with no adverse events in the case of this patient [27,31]. Despite this, the craniotomy required for implantation carries a 1–2% risk. Gliosis and natural scar formation may reduce signal quality over longer periods [23,27]. The Courtine group has advanced WIMAGINE’s integration with personalized spinal electrode arrays and achieved personalized stimulation protocols [12,13,32]. By bridging cortical signals to spinal circuits, useable walking function is generated [17,33]. The Courtine group has achieved clinical translation with regulatory approval for clinical trials in Europe with a proven model for scalable, patient-specific BSIs. The potential for upper limb and autonomic control through the WIMAGINE device makes it a potential cornerstone of neuromodulation for SCI [30,31].

**Table 1:** Comparative analysis and industry leadership predictions for implanted brain–computer interfaces in SCI rehabilitation.

Device	SCI-Specific Application	Decoding Accuracy	Complication Profile	Regulatory Status	Scalability	5-Year Leadership Prediction	Prediction for Adoption (Based on Current Parameters)	References
Neurorestore WIMAGINE-based BSI	Proven efficacy in restoring locomotion; integrates with spinal stimulation for walking and stepping in chronic tetraplegia	80–90%	Zero device-related adverse events; 1–2% craniotomy-related risks (e.g., CSF leakage, gliosis)	Approved for European trials (NCT02550522); potential FDA Breakthrough by 2027	High, but limited by invasive craniotomy	Likely to lead due to ongoing trials and regulatory progress	Strong contender, but may be surpassed by less invasive options	[27,31,32]
Neuralink N1 Implant	Limited SCI data; focused on upper limb prosthetics, potential for future SCI applications	95% (preclinical)	5% electrode detachment, 1% infection risk	Early-stage, faces ethical and transparency hurdles	High potential via robotic implantation, but SCI data lacking	Unlikely to lead without SCI-specific trials	Potential if ethical/regulatory issues resolved	[34,35]
Synchron Stentrode	Growing SCI relevance; prosthetic arm control, pending locomotion trials	85%	3% thrombosis, 2% signal degradation	FDA Breakthrough Device designation (2021)	High due to minimally invasive endovascular approach	Competitive, but limited by precision	Could lead if scalability and precision improve	[36,37,38]
Blackrock Utah Array	Reliable for hand/arm function; limited SCI locomotion data	90%	4% infection, 5% electrode degradation	FDA-approved for investigational use	Low due to invasiveness and cost	Unlikely to lead due to scalability issues	Outdated for scalable SCI solutions	[38,39,40,41,42,43]

### 4.2. Neuralink N1 Implant

The Neuralink N1 iBCI has a large advantage in electrode density, with 1024 electrodes per array, and has been developed along with robot-assisted minimally invasive surgical techniques with high precision [34]. While this advanced implant has demonstrated 95% motor intention decoding accuracy in animal models, its initial trials have not been aimed at restoring walking following SCI. However, the developers recognize this possible alternative use due to the accuracy of the device in decoding motor intentions for limb motor control. A patient (NCT03898323) with tetraplegia and SCI has been able to control a prosthetic arm with the device in the setting of a clinical trial, which is a promising development for the technology. However, the common risks associated with craniotomy and foreign body implantation remain; additionally, a 5% electrode detachment rate is an issue that needs to be addressed. The N1’s future role in restoring motion in SCI remains yet unpublished and without regulatory approval. The innovative robotic implantation techniques and the proprietary electrode technology show potential for scalability and impedance to approval and clinical translation simultaneously. Early research highlight its scalability potential but note ethical concerns around data privacy and long-term safety [34,35].

### 4.3. Synchron Stentrode

Synchron has borrowed the stent from interventional radiologists and endovascular surgeons to create the Stentrode, which is equipped with electrodes and utilizes the transjugular approach to implant electrodes into the brain. This allows it to insert an iBCI into the brain without the need for craniotomy [36]. Clinical trials (NCT05035823) in patients with amyotrophic lateral sclerosis (ALS) and SCI showed 85% accuracy in decoding motor intentions for digital device control, with one SCI patient achieving prosthetic arm movement [36,37]. The question remains whether the introduction of endovascular interventions with complications, including a 3% risk of thrombosis and 2% signal degradation due to vessel occlusion with the requirement of anticoagulant therapy, justifies the moderate success and signal quality. Despite this, preclinical studies in sheep animal models have demonstrated feasibility for spinal interfacing with no human data to date, but the Stentrode gained FDA Breakthrough Device designation in 2021, giving it a head start for potential clinical adoption. Landmark publications emphasize its safety and scalability, but lower channel counts (16 electrodes) limit precision compared to WIMAGINE or Neuralink [36,38].

### 4.4. Blackrock Neurotech Utah Array

The Blackrock Neurotech Utah Array iBCI is a 96-electrode microelectrode array well known to researchers for high-resolution cortical recording. Due to its availability and reliability, it is widely used in research and early clinical trials [38]. Similarly to other devices requiring cortical contact, it is implanted via craniotomy and has enabled precise motor decoding in SCI and ALS patients, with a restoring hand and arm function in tetraplegia via prosthetic control (90% accuracy) with proof-of-concept research ongoing within the BrainGate2 clinical trial [38,39,40,41,42,43]. Unfortunately, its integration with spinal stimulation is limited, with animal models showing partial stepping control when paired with epidural stimulation [17]. Estimations of complications include a 4.5% infection rate, 3% pulse generator malfunction, and other issues affecting long-term signal quality extrapolated from the literature. Due to its extensive research history and FDA approval for investigational use, it is a relevant player and available to researchers in this field, but its invasive implantation protocol and cost limit scalability [42]. Landmark publications highlight its reliability but accept the challenges in transitioning to routine clinical use due to surgical complexity associated with implantation (tedious electrode placement and cable connection to a cranial pedestal), leaving it in a category associated with research applications and animal models rather than clinical translation [40,41].

### 4.5. FDA-Approved Pathways for Clinical Adoption of Implanted Cortical Devices

The WIMAGINE-based BSI, led by the Courtine group, holds the most potential for rapid clinical adoption due to its SCI-specific success and regulatory traction. Evidence-based pathways include the following:

1. Leveraging Breakthrough Device Designation: Apply for FDA Breakthrough status, as Synchron did, to expedite review given WIMAGINE’s demonstrated safety and efficacy [37]. Existing European trial data (NCT02550522) can support this [27].
2. Expanded Access Programs: Use FDA’s Expanded Access pathway to provide WIMAGINE to SCI patients outside trials, generating real-world evidence while awaiting full approval [44].
3. Modular Approval Strategy: Seek FDA approval for WIMAGINE’s cortical component separately from spinal stimulators, leveraging existing approvals for epidural devices (e.g., Medtronic’s Intellis) to streamline the BSI system [8].
4. Collaborative Trials: Partner with US institutions (e.g., Shirley Ryan AbilityLab) to conduct multicenter trials, diversifying patient cohorts and accelerating FDA data requirements [31].

These strategies, grounded in WIMAGINE’s peer-reviewed outcomes and regulatory precedents, can bring it to clinical practice within 3–5 years, addressing SCI’s unmet needs faster than competitors.

## 5. Spinal Surgical Considerations

Spinal surgery for neuromodulation devices in chronic SCI patients is complicated by extradural and intradural scar tissue and adhesions, requiring advanced microsurgical techniques to minimize complications [29]. Stable electrode fixation is critical in the dynamic spinal environment to prevent migration and ensure consistent neuromodulation [30]. Surgical planning must be tailored to the patient’s injury profile, using intraoperative electrophysiological feedback to optimize outcomes [31]. More advanced systems with modular stimulation surfaces may compensate for electrode migration to some extent [31]. Cost-effective surgical protocols and standardized instrumentation are essential for scalability in diverse healthcare settings [26]. Intraoperative neuromonitoring and expertise in anatomic electrode placement are required, with implantation feasible in standard spinal surgical settings and management of complications achievable [26]. Bioengineered scaffolds could provide anchorage and scar control, while modern modular electrodes with adaptive technology may maintain stimulation during spinal movements, improving therapy and outcomes [23,30]. Durable, cost-effective manufacturing and surgical protocols are crucial for global accessibility [26]. Postoperative device failure and surgical complications require management by experienced surgeons [29]. Personalized medicine, including 3D-printed scaffolds or traditional fusion methods tailored to individual spinal anatomy, can enhance electrode placement precision and reduce surgical morbidity, supporting scalable, patient-centered care [32].

## 6. Advancements in Spinal Stimulation Technology

The evolution of spinal stimulation technologies has transformed SCI rehabilitation by targeting lumbosacral circuits to restore motor, sensory, and autonomic functions. Epidural spinal cord stimulation (ESCS) and transcutaneous stimulation have evolved from pain management to functional restoration, leveraging residual neural pathways to bypass lesions. This section analyzes key spinal stimulation devices, focusing on their technological advancements, clinical outcomes, complication profiles, and integration with brain–computer interfaces (BCIs) for SCI.

### 6.1. Courtine Group Epidural Spinal Electrode Arrays

The Neurorestore and Onward Medical ESCS systems are at the forefront of SCI rehabilitation, particularly when integrated with the WIMAGINE-based BSI. These 16–32-channel arrays, implanted epidurally over the spinal cord, deliver spatially selective, synchronized pulses to activate locomotor circuits, guided by real-time machine learning algorithms to coordinate muscular activation for walking [12,13,32]. In a landmark clinical trial (NCT02550522), the arrays were paired with WIMAGINE cortical implants, restoring natural walking and independent stepping within months, with sustained gains over 2 years [27,30]. Preclinical studies in primates validated the arrays’ ability to restore weight-bearing locomotion by targeting dorsal root entry zones, mimicking physiological muscle activation [17,45]. The group’s personalized stimulation protocols, informed by biomechanical modeling and proprioceptive feedback, enhance outcomes by adapting to patient-specific injury profiles [13,46]. Complications are comparable with routine spinal procedures, with a 1–2% risk of postoperative seroma or transient radiculopathy, mitigated by intraoperative neuromonitoring and modular electrode designs [30,47]. The arrays’ relevance lies in their SCI-specific design, regulatory approval for European trials, and synergy with cortical iBCIs, positioning them as the gold standard for BSI-integrated neuromodulation [31,48]. Ongoing trials (NCT04564599) are expanding applications to bladder control and upper limb function, with commercialization planned via Onward Medical’s ARC-IM system [31].

### 6.2. Medtronic Intellis Spinal Cord Stimulation Platform

The Medtronic Intellis platform, an FDA-approved ESCS system, is widely used for chronic pain but has potential to be adapted for SCI rehabilitation in research settings. It is available with a 16-electrode paddle or percutaneous lead, and it delivers programmable stimulation to the thoracic or lumbar spinal cord, with adaptive algorithms adjusting pulse parameters [49]. Complication rates are low, with 3% infection and 2% electrode migration reported, managed by minimally invasive revisions. The platform’s high battery life (up to 9 years) and MRI compatibility enhance its clinical utility, but its generic design limits SCI-specific precision compared to Courtine’s arrays. Its relevance lies in its widespread availability and regulatory approval (FDA 510(k) clearance), making it a practical option for off-label SCI trials with certain elements of the hardware and external design being strikingly similar [27,49]. 

### 6.3. Abbott Eterna Spinal Cord Stimulation System

Abbott’s Eterna SCS system is the smallest FDA-approved implantable stimulator and uses 8–16 electrode leads to deliver low-dose burst or tonic stimulation to the spinal cord. Also designed for pain management, it has been repurposed for SCI rehabilitation, with a 2024 trial reporting improved standing and assisted walking in three patients with incomplete SCI (65–75% success rate). Complication rates are minimal, with 1% infection and 1.5% lead migration, attributed to its compact design and secure anchoring. The system has a wireless programming format and patient-controlled adjustments enhance usability, but its lower channel count and non-SCI-specific algorithms limit precision for locomotion. Its relevance stems from its FDA approval (2022) and potential for rapid adoption in SCI trials due to established infrastructure and existing market. Landmark publications highlight its patient-centered design but underscore the need for SCI-specific stimulation protocols [50,51,52,53].

### 6.4. Boston Scientific Spectra WaveWriter SCS System

The Boston Scientific Spectra WaveWriter combines tonic, burst, and high-frequency stimulation via 16–32 electrode contacts, offering more flexible programming for spinal cord stimulation. Complications include 2% infection and 3% lead migration. The system’s multi-waveform capabilities improve patient-specific outcomes, but its complexity increases programming time, limiting scalability. It also has FDA approval (2018) and potential for SCI adaptation. Landmark publications note its versatility but call for advanced algorithms to target locomotor circuits more specifically [54].

### 6.5. Comparative Analysis and Industry Leadership Predictions

The Courtine group’s epidural arrays lead in SCI-specific applications due to their high success rates (85–95%), zero device-related adverse events, and seamless BSI integration, as evidenced by peer-reviewed trials [27,30,31,45,55,56]. Their personalized, machine learning-driven protocols and regulatory progress (European trials, NCT04564599) position them as the current gold standard. Medtronic’s Intellis, Abbott’s Eterna, and Boston Scientific’s WaveWriter and other models offer robust, FDA-approved platforms with moderate success but lack SCI-specific designs, limiting their precision [30,49,50,51,52,53,54]. In 5 years, the Courtine group’s arrays are likely to dominate, driven by ongoing trials, potential FDA Breakthrough designation by 2027, and commercialization via Onward Medical [31]. In 10 years, Medtronic’s Intellis could gain ground if adapted with SCI-specific algorithms, leveraging its established infrastructure and global reach [30]. Abbott and Boston Scientific are less likely to dominate due to generic designs. The Courtine group’s focus on SCI and BSI integration gives it a long-term edge unless competitors develop tailored locomotor solutions.

### 6.6. FDA-Approved Pathways for Clinical Adoption for Spinal Arrays

The Courtine group’s ONWARD epidural arrays, integrated with the WIMAGINE BSI, hold the greatest potential for rapid clinical adoption due to their SCI-specific efficacy and regulatory traction. Evidence-based pathways for all devices include the following:

1. Breakthrough Device Designation: Pursue FDA Breakthrough status, leveraging European trial data (NCT02550522) and peer-reviewed outcomes [27,30], following Synchron’s precedent [36,37,38].
2. Modular Approval Strategy: Seek separate FDA approval for the spinal arrays, building on existing ESCS approvals (e.g., Medtronic Intellis), to streamline BSI system clearance [8,52].
3. Expanded Access Programs: Use FDA’s Expanded Access pathway to provide arrays to SCI patients outside trials, generating real-world evidence to support full approval [42].
4. Multicenter US Trials: Partner with US institutions (e.g., Craig H. Neilsen Rehabilitation Hospital) to conduct diverse trials, accelerating FDA data requirements [31].

These strategies, grounded in the arrays’ clinical success and regulatory precedents, can bring them to routine practice within 3–5 years, outpacing competitors by addressing SCI’s unmet needs.

## 7. The Bridge

The bridge between cortical implantable brain–computer interfaces (iBCIs) and spinal stimulation systems forms the brain–spine interface (BSI), a transformative approach to restoring locomotion in spinal cord injury (SCI). By decoding motor intentions from cortical signals (e.g., via WIMAGINE’s 64-channel ECoG) and translating them into spatially selective epidural spinal stimulation (e.g., Courtine’s 32-channel arrays), BSIs enable physiological walking [12,27,55]. Real-time machine learning optimizes signal flow, adapting stimulation to patient-specific injury profiles, as demonstrated in trials restoring overground walking [27]. Multi-waveform spinal stimulation enhances flexibility for BSI integration. This synergy, validated in primates and humans, underscores BSIs’ potential to revolutionize SCI rehabilitation, pending scalable, regulatory-approved implementations [12,55].

## 8. Neurological Considerations

Patient selection is pivotal for successful neuromodulation protocols, particularly for novel treatments. Diffusion tensor imaging (DTI) enables precise mapping of residual neural pathways, guiding patient and implant selection based on variable stimulation strategies [57]. Younger patients with greater neuroplasticity achieve better outcomes in reorganizing spared circuits, while older patients with chronic paraplegia face adaptation challenges which can be overcome by improving sensory feedback [58]. Preserving proprioceptive function is essential for smooth walking, requiring stimulation protocols that integrate proprioceptive nuances [19]. Restoring walking through neuromodulation demands personalized medicine, with machine learning optimizing outcomes in real-time, ensuring unique stimulation protocols for each patient [25]. Targeting spinal and supraspinal circuits may maximize neuroplasticity and motor recovery [34]. Ethical challenges arise with personalized stimulation, as machine learning requires data-sharing frameworks governed by strict ethical guidelines to protect patient privacy [25]. Patients’ diverse expectations and quality-of-life definitions necessitate clinical management of goals and priorities [26]. Selection criteria must consider health parameters influencing neural plasticity and the potential for sensory feedback via iBCIs to enhance motor recovery [20,59].

## 9. Postsurgical Multimodal Neurorehabilitation

Long-term outcomes, device durability, therapy effectiveness, and multimodal neurorehabilitation tailored to complex device use are critical for meeting patient needs. Neuromodulation combined with neurorehabilitation enhances motor function and neural circuit reorganization [60,61]. Occupational and physical therapy remain lifelong components for paraplegic patients’ health, requiring community resources to support this population [62]. Integrating physical, occupational, and psychological therapies addresses SCI patients’ holistic needs [63]. Remote platforms with wearable sensors enable continuous monitoring and therapy adjustments, reducing healthcare system burdens [46]. In cases of residual neurological function, active physical therapy synergizes with neuromodulation, leveraging existing frameworks to manage device recipients [60,62]. These platforms lighten SCI’s biosocial impacts by reducing reliance on costly medical services, fostering community-based care, and enhancing accessibility [7,26]. Combining BCIs with physical therapy and neuromodulation offers a future for combinatorial therapies, improving neural circuit remodeling and rehabilitation outcomes using established structures [21,47].

## 10. Bench-to-Bedside Medicine and Surgery: Technology for Patients

Translating neuromodulation from bench to bedside requires integrating scientific innovation, clinical expertise, and ethical oversight to achieve meaningful patient outcomes. Preclinical studies demonstrate brain–spine interface efficacy in primate models, using intracortical microelectrodes and epidural stimulation to bypass spinal lesions [17]. Clinical trials validate these findings, with advances in restoring overground walking and independent stepping in chronic SCI patients [3,50]. Scalability challenges arise from system complexity and experimental nature, necessitating specialized surgical expertise and controlled rehabilitation settings [8,62]. Simplifying implants and surgical protocols into modular designs can enhance adoption [26]. Leveraging technology from existing implants for other indications can expedite approvals while maintaining safety standards, supported by long-term data [8,23]. Patient selection requires transparent clinical and radiological criteria, such as DTI-identified viable pathways [57]. The commitment to neurological gains must be clearly communicated, with clinician-driven management of expectations [26]. Addressing global healthcare disparities requires international oversight to ensure equitable training and reimbursement, preventing neuromodulation from being limited to high-income countries [7]. Feedback from SCI communities, both automated and voluntary, is critical to align technological advancements with real-world needs, prioritizing quality of life and independence [39]. Interdisciplinary collaboration among neuroscientists, engineers, and clinicians is essential to refine stimulation algorithms using machine learning for real-time personalization [25]. Industry partnerships can drive cost-effective, scalable devices, while global clinical trials validate efficacy across diverse populations [8]. Embedding ethical principles—equity, transparency, and patient empowerment—into development ensures neuromodulation transitions from a research tool to a transformative clinical therapy, offering hope to millions with SCI worldwide [3,7,26].

## 11. Discussion

The integration of neuromodulation and digital brain–spinal cord interfaces represents a paradigm shift in SCI treatment, moving beyond compensatory strategies toward restoring physiological function. This review highlights exponential progress in neuromodulation, evidenced by clinical trials demonstrating overground walking and independent stepping in chronic SCI patients [3,30]. Unlike stem cell therapies or immune modulation, which face significant translational hurdles [1,2], neuromodulation leverages residual neural pathways to bypass lesions, offering a practical solution using clinically tested methods [11,12,13]. Realizing this technology’s potential requires addressing challenges in surgical precision, patient selection, rehabilitation, and ethical implementation.

Surgically, implanting iBCIs and spinal electrodes demands neurophysiological and anatomical precision, with robot-assisted stereotaxy and intraoperative neuromonitoring becoming fixed requirements for targeting motor and sensory cortices or spinal circuits [22,27]. Biocompatible materials and minimally invasive approaches, such as endovascular stent electrodes, mitigate risks like gliosis and infection, but standardization is critical for safety and scalability, as these techniques lack extensive long-term data and may introduce undocumented risks [23,28]. Spinal surgeries in chronic SCI, complicated by scar tissue and adhesions, could benefit from bioengineered scaffolds and modular electrode designs, enhancing outcomes by stabilizing implants and reducing morbidity [30,31,32]. Global training programs, potentially using virtual reality platforms, are essential to equip neurosurgeons and spine surgeons for these procedures across diverse healthcare settings [8].

There is the problem of the incredible inhomogeneity that characterizes patients with neurological injuries, which can encompass SCI within 1–2 years of initial injury to chronic SCI patients with injuries that are many years old. Depending on the degree of paralysis according to the American Spinal Injury Association Score (AISIA), chronic SCI patients could, in future treatments, be selected for this type of procedure, especially in the case of incomplete injury [48]. This can add to the level of complexity and inhomogeneity of cohorts. This also means that expectations and outcomes will vary even more extremely, and motor gains will be incrementally calibrated, to the point where certain cohorts will attain locomotion while others will only achieve unassisted transfer. The risks of surgeries and the invasiveness of the technique will have to be adjusted to manage both small and large motor gains; for this reason, a diversity of surgical outcomes benefits from a spectrum of surgical invasiveness. The preliminary findings of Professor Jia Fumin’s team at Fudan University’s Institute of Science and Technology for Brain-Inspired Intelligence were excluded from this comparative analysis; however, these reports bear mention due to promising results and the reported very minimally invasive procedure with proportionately high motor gains. This type of procedure can potentially open the door to a very large spectrum of total and sub-total SCI patients [64,65].

Patient selection is critical for optimizing neurological outcomes, with DTI guiding identification of candidates with viable neural pathways [57]. Neuroplasticity, particularly in younger patients, and proprioceptive feedback integration are vital for motor recovery [19,58]. Machine learning-driven stimulation protocols provide personalized approaches, adapting to individual neural responses, but ethical implementation requires robust data governance to protect privacy and ensure reproducibility [25,44]. Rehabilitation, a cornerstone of recovery, must incorporate activity-based therapies, tele-rehabilitation, and community-based support to sustain gains and address biosocial challenges [60,61,62,63]. Combinatorial therapies integrating neuromodulation with active physical therapy and BCIs show synergistic effects, enhancing neural circuit remodeling and outcomes [21,47].

Ethically, transitioning to routine clinical use must prioritize equity and patient empowerment. Informed consent, long-term management, and evidence-based ethical guidelines are essential given surgical risks and intensive rehabilitation [26]. Global healthcare disparities necessitate partnerships with international organizations to establish infrastructure in low-resource settings, ensuring neuromodulation is not limited to high-income nations [7]. Patient-centered design, informed by SCI community feedback, aligns advancements with real-world needs, prioritizing independence and quality of life [39]. Regulatory frameworks must balance expedited approvals with rigorous safety standards, using long-term trial data to build trust among patients, clinicians, and healthcare systems [8,44].

The field must embrace interdisciplinary collaboration to overcome logistical and scientific barriers. Neuroscientists, engineers, and clinicians should refine stimulation algorithms and develop cost-effective devices, with industry partnerships driving commercialization [9,25]. Global clinical trials, inclusive of diverse populations, will validate efficacy and address underrepresentation [28]. Restoring function raises ethical questions about societal roles and patient identity, requiring ongoing dialogue within frameworks like the Belmont Report [66]. By embedding equity, transparency, and patient agency into development, neuromodulation can transform SCI care, delivering tangible functional restoration to millions worldwide. This vision demands concerted efforts to bridge bench-to-bedside gaps, ensuring technological promise translates into meaningful, accessible, and ethical clinical outcomes.

## 12. Conclusions

The emergence of realizable systems for restoring motor functions following SCI is a long unrealized goal of the medical field as a whole and reviewing the literature makes a forward-thinking attitude particularly exciting because the potential exists for paralyzed patients globally to walk again within this generation. Interdisciplinary cooperation, possibly new regulatory bodies, and a new era of inter-industry/medical inter-cooperation will be necessary for these goals to reach the corners of society that need this neuromodulation technology the most. The transformation for paraplegic patients and the field of neurorehabilitation is limitless and presents new ethical boundaries that physicians and researchers alike will need to explore.
